# The mediating role of self-continuity on the link between childhood adversity and loneliness in later life

**DOI:** 10.3389/fpsyg.2022.1039504

**Published:** 2022-11-21

**Authors:** Charikleia Lampraki, Daniela S. Jopp, Dario Spini

**Affiliations:** ^1^Institute of Psychology, University of Lausanne, Lausanne, Switzerland; ^2^Swiss National Centre of Competence in Research LIVES, University of Lausanne, Lausanne, Switzerland; ^3^Institute of Social Sciences, University of Lausanne, Lausanne, Switzerland

**Keywords:** critical life events, childhood adversity, divorce, bereavement, identity, life course

## Abstract

Many factors may influence adaptation to critical life events such as divorce and bereavement in the second half of life, including having faced childhood adversity. However, pathways to reduced adaptation success are poorly understood. Self-continuity, an identity mechanism that incorporates life changes into a coherent life story, may contribute to better adaptation to adult critical life events, such as feeling less socially and emotionally lonely. We investigated the mediating role of self-continuity channeling the effects of childhood adversity on later life adaptation outcomes among individuals who had experienced divorce or bereavement. Data were derived from the longitudinal LIVES Intimate Partner Loss Study conducted in Switzerland from 2012 to 2016. The sample consisted of individuals who had experienced divorce (*n* = 416, *M_age_* = 57.35) or bereavement (*n* = 339, *M_age_* = 71.36) in later life, and a continuously married control group (*n* = 925, *M_age_* = 67.04). Multilevel moderated mediations were used. Self-continuity mediated the effect of childhood adversity on emotional loneliness for all marital groups, but to a greater extent among divorcees. Self-continuity also mediated the effect of childhood adversity on social loneliness; however, this effect did not differ by marital group. In conclusion, childhood adversity was associated to greater loneliness in later life through self-continuity. Divorcees were the most impacted group regarding emotional loneliness, as they experienced lower levels of self-continuity. Interventions that aim to reinforce self-continuity may help overcome social and emotional loneliness, especially for individuals who have experienced the loss of their partner through divorce.

## Introduction

Critical life events throughout the life course can negatively impact identity and mental health (Spini et al., 2017): they can lead to identity confusion (Slotter and Emery, 2017), feeling less satisfied with life (Lucas, 2007), lonelier (Dykstra and de Jong Gierveld, 2004; Nicolaisen and Thorsen, 2014) or even becoming depressed (McLaughlin et al., 2010). As individual adaptation pathways to critical life events may differ (Pudrovska and Carr, 2008; Perrig-Chiello et al., 2014) and require a better understanding of the processes that take place, identity may play an important role in explaining how early life adversity is linked to adaptation to later life events. For instance, it is well documented that adverse childhood events not only influence a child’s well-being but have long-lasting effects in later adulthood (Turner and Lloyd, 1995; McCarthy and Maughan, 2010; Nurius et al., 2015; Cheval et al., 2019), such as loneliness (Nicolaisen and Thorsen, 2014). Therefore, adapting to critical life events that occur later in life, such as divorce or spousal bereavement, may be more challenging for individuals who have experienced adversity during childhood, especially if these early events jeopardized the development of close relationships to family or other social partners.

**Figure 1:**
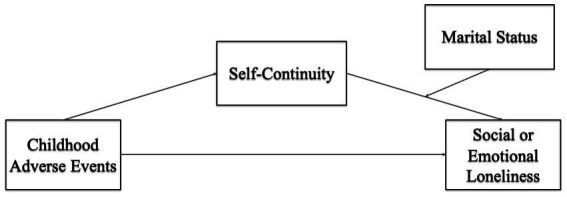
Hypothesized model of the moderated mediation.

Another pathway through which childhood adversity can exert a long-term effect is its negative influence on identity processes (Grotevant et al., 2017; Markovitch et al., 2017), such as self-continuity (Lampraki et al., 2022). More specifically, individuals may be more vulnerable for later critical life events, if their self is less well defined, as a consequence of childhood adversity. Self-continuity is an identity process that emerges in adolescence as a positive developmental outcome (Erikson, 1968) and continues to develop across the life course, providing “a sense of connection between one’s past and one’s present” (Sedikides et al., 2016). However, little is known regarding the role that self-continuity may have on the link between childhood adversity and the adaptation to frequent critical life events, such as partner loss, and more specifically, important maladaptive outcomes, such as social and emotional loneliness. In this study, we aim to identify to what extent childhood adversity presents a risk factor for later-life social and emotional loneliness, especially in the context of partner loss. Consequently, we investigate whether self-continuity is a possible mediator of the relationship between childhood adversity and loneliness for individuals who have experienced divorce or spousal bereavement in the past 5 years, using a group of continuously married individuals as reference. Specifically, we expect that more childhood adversity will relate to lower levels of self-continuity, which in turn will be associated to higher levels of social and emotional loneliness, and that this relationship will vary in magnitude for marital status groups (Figure 1).

In the context of this study, it is important to understand how self-continuity develops. Self-continuity emerges from the temporal comparison (past- or future-directed comparisons) of core self aspects that constitute a meaningful whole, distinguishing individuals from others and giving them a sense of uniqueness. It represents the temporal facet of the self, along with self-concept clarity, which is the structural facet of self; together they are viewed as two basic components of identity (Gregg and Sedikides, 2018; Jiang et al., 2020). Past self-continuity has been positively related to psychological well-being (McAdams et al., 2001) and physical health (Anderzén and Arnetz, 1999), and negatively to loneliness (Van Tilburg et al., 2019), to suicide (Chandler and Proulx, 2008), and to life events such as job loss (Sadeh and Karniol, 2012). Nevertheless, research is still limited regarding the factors that influence the development of self-continuity. According to Sedikides et al., (2015), a protective factor that seems to enhance self-continuity is nostalgia. However, having memories that are painful to recall—such as childhood trauma or adversity—may impede individuals from experiencing a strong feeling of self-continuity. Consequently, childhood adversity was found to be related to lower levels of self-continuity in later life, and divorcees seemed to be more impacted than widowed or married individuals (Lampraki et al., 2022). These findings suggest that a difficult childhood can challenge the ability to incorporate memories from the past into a coherent life story and to maintain a clear and time-persistent image of the self.

Having a less clear sense of who they are and who they were, individuals have difficulties in engaging in the temporal self-comparisons that reinforce self-continuity. Being uncertain of who they are, whether and how they have changed, they may also question other aspects of their lives, e.g., re-evaluating their need for romantic, friendly, or family connection with others. If individuals with childhood trauma feel less self-continuity, do they also exhibit poorer adaptation outcomes (e.g., higher loneliness) when experiencing partner loss? Thus, it is important to highlight the impact of critical life events and adversity at different life moments on identity and how their accumulation can contribute to increasing vulnerability, which can be manifested as social or emotional loneliness.

For instance, critical life events are considered triggers of change throughout the life course with ambivalent consequences for individuals. However, when these events occur during important developmental stages, such as in childhood or adolescence, they can have stronger and perhaps longer-lasting effects on identity processes and well-being. Childhood adversity, as defined by McLaughlin, (2016), refers to “experiences that are likely to require significant adaptation by an average child and that represent a deviation from the expectable environment.” Poverty, physical and emotional neglect, and witnessing or suffering from physical or sexual violence in childhood are adverse experiences with well documented negative consequences on children’s development and well-being. According to Nurius et al. (2015), childhood adversity can also exacerbate the effects of subsequent life course stressors and impede adaptation and coping to later adverse events across the life course. In line with these findings, neurophysiological research has related early-life adversity with permanent changes in the brain structure and the hypothalamic–pituitary–adrenal axis, causing higher stress reactivity and reduced cognitive functioning in adult life (Lupien et al., 2009; Hanson et al., 2016). Individuals who faced adverse childhood experiences have also been found to develop poor attachment styles that lead to negative relationship patterns in adulthood (McCarthy and Maughan, 2010) and tend toward poor marital outcomes, such as low relationship quality or divorce (Whisman, 2006).

According to Bowlby (1951), who developed the theory of attachment, loneliness is one of the outcomes of insecure attachment in early life stages between the mother and the infant, highlighting how adverse childhood experiences (e.g., emotional neglect) may have a distal link to adult loneliness. Based on the theory of mother–child attachment, Weiss’s theory (Weiss, 1973) distinguishes loneliness in social and emotional: Social loneliness is linked to an unengaging social environment and to a lack of friends or family, who, if present, could act as sources of social support and provide the individual with a sense of connectedness. Lack of social embeddedness is also related to feeling excluded, bored and without purpose. Emotional loneliness, instead, is associated with the absence of a significant other, such as a spouse, and is closely related to anxiety, lack of a sense of security, and aloneness. Finally, both types of loneliness emerge when a person’s need for connectedness is unmet.

Loneliness, similar to feeling dissatisfied with life or depressed, is not only related to early life stressors (e.g., poor mother–child attachment) but can become pronounced when facing new coping challenges. The loss of one’s spouse can be particularly taxing for an individual’s well-being (Dykstra and de Jong Gierveld, 2004; Pudrovska and Carr, 2008; Nicolaisen and Thorsen, 2014), as such an event requires a reevaluation of one’s central social roles and identity aspects (Slotter and Emery, 2017). For instance, experiencing self-discontinuity may trigger a reevaluation of the need for social connectedness, resulting to perceiving the existing social partners as non-adequate or non-supportive which then can lead to increased social loneliness and decreased social continuity (Atchley, 1989), too. Zhou et al., (2008) found that lonely individuals who used more nostalgia, a key process in restoring self-continuity (Sedikides et al., 2015), perceived higher levels of social support. Therefore, it is not only the reduction of social partners (or the absence of a romantic partner) that can result in increased feelings of loneliness, but also internal processes that evaluate the existing social ties (or romantic relationships) as not sufficient to satisfy the need for connectedness or support after a critical life event. Even though research has documented the lasting impact of adverse childhood events on later life psychological processes, such as loneliness (Nicolaisen and Thorsen, 2014), due to underlying physiological, social, and personal mechanisms, little is known regarding the role of identity processes, such as self-continuity, in the context of adaptation to intimate partner loss.

Adaptation to critical life events, such as divorce and bereavement, is achieved when individuals regain their pre-event levels of well-being, representing a process that needs time (Lucas, 2007). This has, for example, been documented for partner loss by Lucas, (2005) who examined trajectories of life satisfaction over a period of 18 years, considering pre- and post-event levels. He found that divorcees tended to recover after some time, and approached their pre-divorce levels of satisfaction with life, however, without completely reaching their baseline level. He also found that divorcees had lower pre-marriage levels of life satisfaction compared to the married. Widow(er)s, similarly to the divorcees, did not reach their pre-widowhood levels of life satisfaction. Moreover, it has also been documented that a strong perception of self-continuity was related to feeling less socially lonely at a later adaptation phase after divorce, but not right after the occurrence of the event (Lampraki et al., 2019). These findings indicate that while adaptation to loss is a matter of timing, and that there are factors that may help adapting better or faster, there are also preceding-to-event factors that differentiate the adaptation outcomes. Based on the theoretical considerations described earlier, interesting candidates may include childhood adversity, as an important precursor or risk factor, as well as self-continuity as a central identity mechanism.
Notably, if the loss of one’s partner occurs in the second half of life, it can be especially critical, given that advancing into older age is associated with other events and limitations, such as health-related [e.g., cardiovascular issues, (Levine et al., 2021); or menopause (Mansikkamäki et al., 2015)] or work-related changes [e.g., retirement (Pilipiec et al., 2021)]. Consequently, individuals are often confronted with new economic, social, and psychological challenges. For instance, continuous engagement in important social groups provides individuals with specific self-facets and roles that, if lost, challenge well-being (Jetten et al., 2012). Particularly in later life when losses in social relations are more common, experiencing divorce or bereavement increases vulnerability, as individuals not only lose the role of the spouse, but often face challenges in the remaining social network: E.g., individuals can experience a reduction in their social network after divorce (Widmer et al., 2012), as friends may choose to support their ex-spouse. During the initial post-loss phase, divorcees often also face social isolation and loneliness due to changes in social embeddedness (de Jong Gierveld et al., 2006). Widow(er)s are likely to increase their level of social engagement in order to compensate for their lost social role and maintain continuity (Utz et al., 2002), and they experience both losses and gains in social partners due to bereavement (Ha, 2008). Van Baarsen (2002) found that pre-loss or increased post-loss social support did not benefit bereaved individuals in the short-term after the death of the spouse. However, increased support reduced social loneliness in the long-term after spousal loss. Even though they receive more support during the initial time after the loss from family and friends, they also feel left alone and have a high risk for depressive symptoms (Golden et al., 2009) and loneliness (Nicolaisen and Thorsen, 2014). However, an important question in adaptation to spousal loss research is whether divorce and bereavement are equally critical as events for one’s wellbeing.
Divorce and bereavement may resemble one another as critical life events, as individuals lose the valued social role of being a spouse; however, the psychological implications for divorce and bereavement differ (Pudrovska and Carr, 2008): For instance, Pinquart, (2003) found that divorcees felt more lonely than bereaved individuals. An important difference is that the dissolution of the marriage through divorce can be a process in which an individual can feel in control: the divorce as the result of a personal decision (i.e., to voluntarily end one’s marriage) is associated to personal control and agency, with better psychological outcomes for the initiators of the break-up (Amato, 2000). Instead, bereavement may involve a lack of control or agency associated to partner’s death; other processes, such as anticipation of the spousal death in case of disease trajectories or acceptance of death have important effects on adaptation outcomes (Infurna et al., 2017). A significant shortcoming in this body of research is the lack of investigation of identity processes, such as self-continuity, which may explain the differential adaptation outcomes, and how these identity processes may promote adaptation.

With this study, we investigated the impact of distal (i.e., childhood adversity) and proximal (i.e., later life partner loss) critical life events on social and emotional loneliness, considering self-continuity as an identity process that channeled the effect of the events. Specifically, we examined whether the links between childhood adversity and social or emotional loneliness were mediated by self-continuity: Although it has been documented that childhood adversity relates to higher loneliness in later life (Nicolaisen and Thorsen, 2014; Lampraki et al., 2022), self-continuity may act as a mediator of this link, as its function is to link present and past experiences and integrate them into a coherent whole (Sedikides et al., 2015). Therefore, we expected that when self-continuity was considered, the link between childhood adversity and social and emotional loneliness would become weaker. Finally, we also expected that the magnitude of the mediation would vary by marital status, in line with previous research showing that divorced, bereaved, and married individuals differ with regard to self-continuity (Lampraki et al., 2022) and loneliness (Pinquart, 2003; Dykstra and de Jong Gierveld, 2004; Nicolaisen and Thorsen, 2014).

## Materials and methods

### Sample and procedure

We used data from the longitudinal LIVES Intimate Partner Loss Study (Perrig-Chiello et al., 2014), conducted in Switzerland (German- and French-speaking parts) from 2012 to 2016 in three waves, at two-year intervals. The full study (*N* = 2,168) aimed at investigating adaptation pathways after later life partner loss and assessed longitudinally a comprehensive set of personal characteristics, social contacts, and support variables, as well as pre- and post-loss relationship conditions and experiences. Participants were mainly recruited through the Swiss Federal Office of Statistics, and a minority through advertisements. Participants had to be currently married for at least 15 years or to have lost their partners through separation/divorce or bereavement after marriage lasting at least 15 years. The sample was stratified by age, gender, and marital status. The present study sample included a total of 1,680 individuals aged 46–92 years old: divorced or separated, with loss during the past 5 years, *n* = 416; widowed during the past 5 years, *n* = 339; and married (*n* = 924). Approval for the study was obtained from the ethics committee of the University of Bern (2011-10-3864). Participants were informed about the study in writing and asked to fill out a paper-and-pencil or online questionnaire. They were not compensated as their participation was voluntary. Finally, they were informed that their data would be treated anonymously and that they could quit the study or choose not to answer any question at any time.

### Measures

#### Dependent variables

We measured social and emotional loneliness with the short de Jong Gierveld Loneliness Scale (De Jong-Gierveld and Kamphuls, 1985). The scale assesses social and emotional loneliness, as suggested by Weiss (1973), with three items for each dimension (e.g., social loneliness: “There are plenty of people with whom I feel closely connected;” emotional loneliness: “I feel a general emptiness”). The participants answered on a 5-point Likert scale (1 = *no* to 5 = *yes*). Negatively worded items were recoded, and mean scores were calculated for both loneliness indicators. Higher values indicated greater loneliness (social loneliness, *α* = 0.89; emotional loneliness, *α* = 0.78; Wave 1).

#### Independent variables

Using a filter question (“Have you ever lost your long-term partner through separation, divorce, or death; and if so, when?”), we created a grouping variable that distinguished the marital status groups into divorced (including separated), bereaved, and married individuals (1 = *separated/divorced*, 2 = *widowed*, 3 = *married*). Sociodemographic variables were measured in all waves and included age, gender (0 = *men*, 1 = *women*), and financial adequacy (1 = *I do not have enough money to support myself* to 3 = *I have more than enough money to support myself*). Time since the event was calculated by subtracting the year of a participant’s loss from the year of questionnaire administration for each wave in the divorced and widowed groups.

We measured the number of important groups (i.e., participants’ social continuity) using the Exeter Identity Transitions Scales (Haslam et al., 2008). Participants reported up to six social groups that they belonged to and indicated how important these groups were to them using a 5-point Likert scale (1 = *not important* to 5 = *very important*). Only social groups that were rated as important or very important were used to construct a sum score for each wave, with a theoretical range from 1 = *one important or very important group* to 6 = *six important or very important groups*. When individuals reported many important social groups, and they maintained them through study waves it indicated higher levels of social continuity, in line with social continuity theory by Atchley, (1989). Examples of social groups included being a member of a sports club, of a political party, of a choir, of an organizing committee etc., among others. The presence of a new partner was measured with a single item in all waves addressing divorced and widowed participants (“Are you currently involved in a romantic relationship?”). The answering format was 1 = *yes* or 0 = *no*.

Childhood adversity was measured once (in wave 3) with six items capturing the frequency of childhood (or adolescent) adversity: (a) “Did you have the feeling that, in your family, no one loved you or thought of you as being someone important or of value?” (b) “Have you been frightened or hurt by a person of reference?” (c) “You did not have enough food to eat, or clean clothes, or you were not cared after when you needed it;” (d) “Have you witnessed violence between your parents?” (e) “Did an adult beat you with an object such as a belt or a stick, kick or burn you?” (f) “Have you been touched by a reference person or authority figure, or have you been forced to sexually touch another person?” The answering format was 0 = *never* to 4 = *very often.* A mean composite score was calculated, with higher values indicating more frequent childhood adversity (*α* = 0.77).

#### Mediator

Self-continuity was measured using three items from the Exeter Identity Transitions Scales [e.g., “With time a lot of things have changed, but I’m still the same person;” (Haslam et al., 2008; Lampraki et al., 2019)]. Answers were given on a 5-point scale (1 = *does not apply to me at all* to 5 = *fully applies to me*). We calculated a mean composite score, with higher values indicating greater self-continuity for each wave. The scale had good internal consistency across study waves (e.g., Wave 1, *α* = 0.81).

### Analytical strategy

We used multilevel mediation models with two level to test whether self-continuity mediated the link between childhood adversity and adaptation outcomes, such as social and emotional loneliness. The first level we represented the different measurement points (i.e., wave 1, wave 2, wave 3) and the second level the person, as this is the best design for repeated measures.

Given that the married, divorced, and bereaved groups had some variables in common, but also others that were only present in the loss groups (e.g., time since partner loss and having a new partner were only relevant for the divorced and bereaved), we conducted models for the total sample, as well as models for the different marital status groups. Specifically, we ran two sets of (second stage) moderated mediation models, using the marital status variable as the moderator of the link between self-continuity and social or emotional loneliness. In the first set, we excluded the time since event and new partner variables, as these were relevant only to divorced or bereaved individuals. In the second model set, we ran (second stage) moderated mediation models using a variable that distinguished only divorced from bereaved individuals as a moderator (married individuals were excluded), adding all relevant variables for the loss groups. The results of these first analyses indicated that the marital status variable moderated the effect of self-continuity on emotional loneliness (Appendix 1, Supplementary Table S2 for the first two models of emotional loneliness). For social loneliness, the (second stage) moderation effect was not significant. Therefore, to identify how the mediation differed across marital status groups, we conducted separate analyses for the divorced, bereaved, and married groups for emotional loneliness.

To test between-subjects differences, we included the person means for age, financial adequacy, time since the event, number of important groups, new partner, and self-continuity, as well as gender and childhood adversity. To test within-subjects variation, we also person-mean centered age, number of important groups, new partner, and self-continuity. In preliminary analysis, we also person-mean centered financial adequacy. However, in order to present the most parsimonious model and focusing on variables that were central to our analysis, we decided to exclude the within-subjects variation of financial adequacy as it did not improve the final model. Fixed-effects estimates, random intercepts, slopes, and covariances were calculated within all groups, but only the final, most parsimonious (i.e., best-fit) models are presented.

To confirm sufficient within-subjects variability, which would justify multilevel modeling, we first ran fully unconditional models (no predictors or covariates included), then added the independent variables. Self-continuity was the last variable included in the mediation model. To determine the between-subjects variability, we calculated the intraclass correlation coefficient (Raudenbush and Bryk, 2002). We used SPSS (Version 27) and the corresponding macro designed for testing multilevel mediation models (Hayes and Rockwood, 2020). Results are presented using unstandardized Beta coefficients. For the mediation analyses, we tested indirect effects using Monte Carlo confidence intervals (MCCIs).

## Results

Descriptive statistics of study variables are presented in Table 1 for the whole sample and for each marital status group, referring to pooled means across the three waves of data collection. Descriptive statistics of the study variables by wave are presented in the Appendix (Supplementary Table S1).

**Table 1:** Descriptive statistics of study variables by marital status.

	(*N* = 1,680)	Divorced (*n* = 416)	Widowed (*n* = 339)	Married (*n* = 925)		
	*M* (SD) or *N* (%)	*M* (SD) or *N* (%)	*M* (SD) or *N* (%)	*M* (SD) or *N* (%)	*F* or *χ^2^*	*p*-value
Social loneliness	0.91 (0.86)	1.08 (1.05)	0.88 (0.92)	0.76 (0.85)	43.96	<0.001
Emotional loneliness	0.75 (0.76)	0.95 (1.00)	0.88 (0.84)	0.52 (0.66)	154.68	<0.001
Age	66.80 (11.44)	57.35 (6.72)	71.36 (8.69)	67.04 (11.10)	730.78	<0.001
Gender (women)	974 (58)	280 (67)	205 (61)	489 (53)	77.75	<0.001
Financial adequacy	2.08 (0.41)	2.01 (0.50)	2.12 (0.43)	2.14 (0.45)	29.49	<0.001
Time since event	4.80 (1.51)	4.09 (2.12)	4.84 (2.06)	-	103.95	<0.001
Number of important groups	0.56 (0.78)	0.72 (0.99)	0.68 (1.04)	0.67 (1.00)	33.37	<0.001
New partner	0.28 (0.39)	0.36 (0.48)	0.18 (0.39)	-	150.57	<0.001
Childhood adversity	1.59 (0.68)	1.77 (0.77)	1.53 (0.62)	1.48 (0.56)	91.99	<0.001
Self-continuity	2.54 (1.00)	1.99 (1.14)	2.56 (1.05)	2.72 (0.95)	256.31	<0.001

Descriptive statistics refer to 3-waves-pooled data (except from childhood adverse events which was collected in wave 3). Time since event and new partner apply only to divorced and widowed individuals. *χ*^2^ test was performed only for gender.

### Moderated mediation analyses

**Figure 2:**
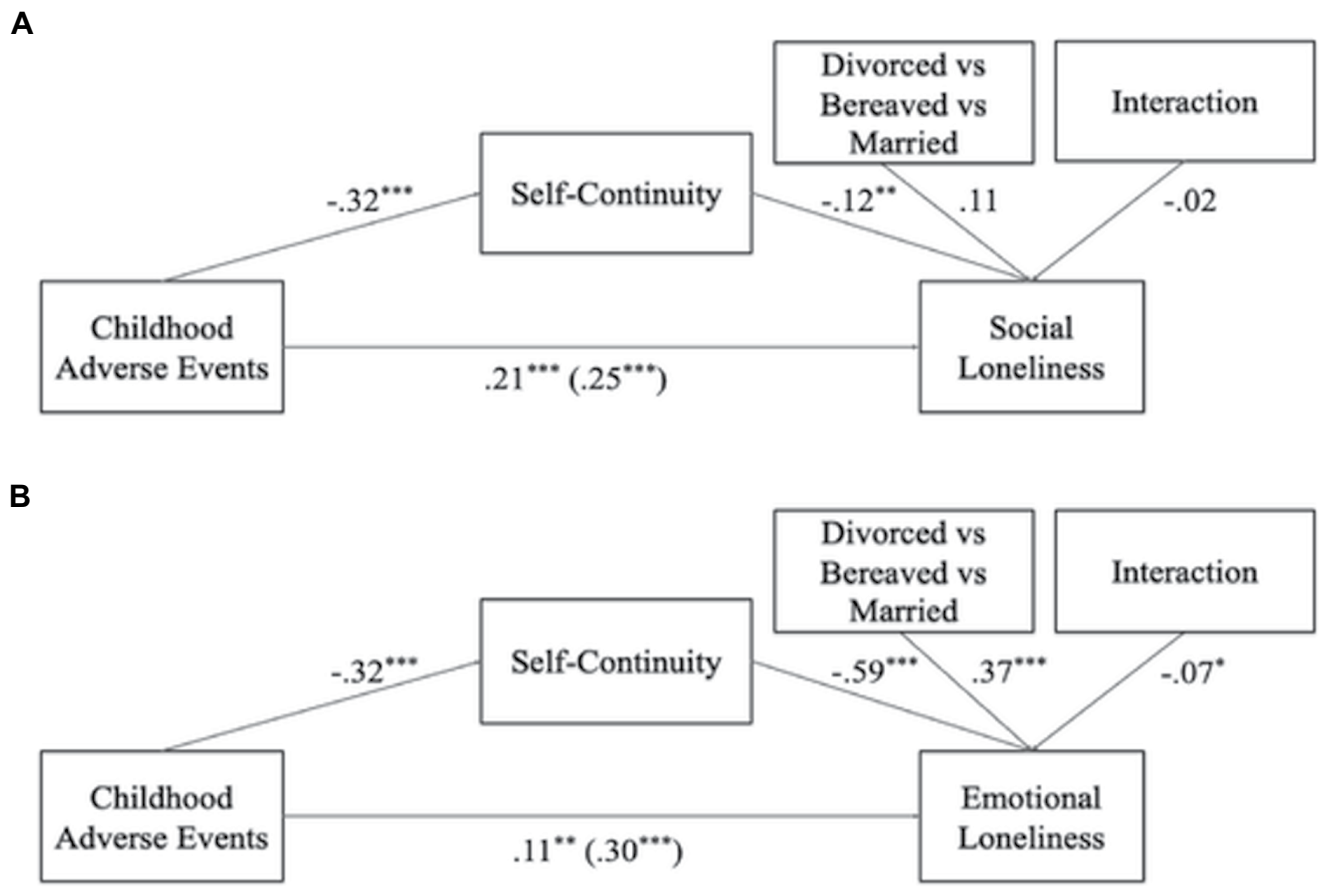
Between-subjects’ moderated mediations of self-continuity on the link between childhood adversity and social and emotional loneliness, with marital status as moderator. Panel **(A)**, indirect effect *B* = 0.04, *SE* = 0.01, 95% MCCI (0.012, 0.069), *z_sobel_* = 2.72, *p* < 0.01; Panel **(B)**, indirect effect *B* = 0.19, *SE* = 0.03, 95% MCCI (0.135, 0.239), *z_sobel_* = 7.03, *p* < 0.05; *p < 0.05; **p < 0.01; ***p < 0.001.

**Figure 3:**
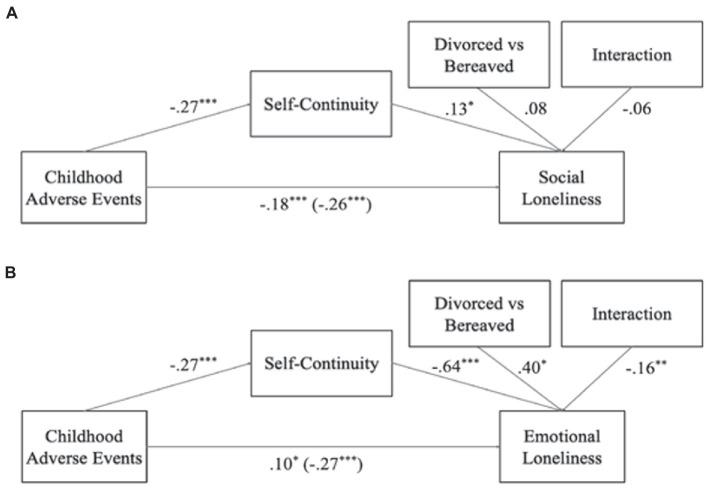
Between-subjects’ moderated mediations of self-continuity on the link between childhood adversity and well-being outcomes, with divorced vs bereaved as moderator. Panel **(A)**, indirect effect *B* = −0.03, *SE* = 0.01, 95% MCCI (−0.057, −0.002), *z_sobel_* = −1.99, *p* < 0.05; Panel **(B)**, indirect effect *B* = 0.13, *SE* = 0.03, 95% MCCI (0.080, 0.186), *z_sobel_* = 4.93, *p* < 0.001; ^+^*p* < 0.10; *p < 0.05; **p < 0.01; ***p < 0.001.

The moderated mediation models tested whether self-continuity mediated the links between childhood adversity and social loneliness (Figure 2A) and emotional loneliness (Figure 2B), respectively, and whether this process was moderated by marital status (i.e., differences between divorced, widowed, and married individuals). In these first models, we only included covariates that applied to all marital status groups (i.e., age, gender, financial adequacy, and important social groups). We found a partial mediation effect of self-continuity on the link between childhood adversity and social loneliness (*B* = 0.04, *SE* = 0.01, 95% MCCI [0.012, 0.069], *z_sobel_* = 2.72, *p* < 0.01). However, the marital status variable showed no moderating effect (B = −0.02, *p* > 0.05), suggesting that individuals with difficult childhoods experienced less self-continuity in later life and felt more socially lonely, regardless of having experienced the loss of their partner or not. To test whether this process differed between divorced and widowed individuals when including loss-related covariates (i.e., time since loss and having a new partner), we repeated the same model, adding these loss-related covariates and distinguishing divorced from widowed individuals as the moderating variable (Figure 3A). In this model we also found a partial mediation effect (*B* = −0.03, *SE* = 0.01, 95% MCCI [−0.057, −0.002], *Z_sobel_* = −1.99, *p* < 0.05) and the moderator had no effect on the mediating process (*B* = −0.06, *p* > 0.05), indicating that even after including the loss-related variables, divorced and widowed individuals’ outcomes did not differ.

**Figure 4:**
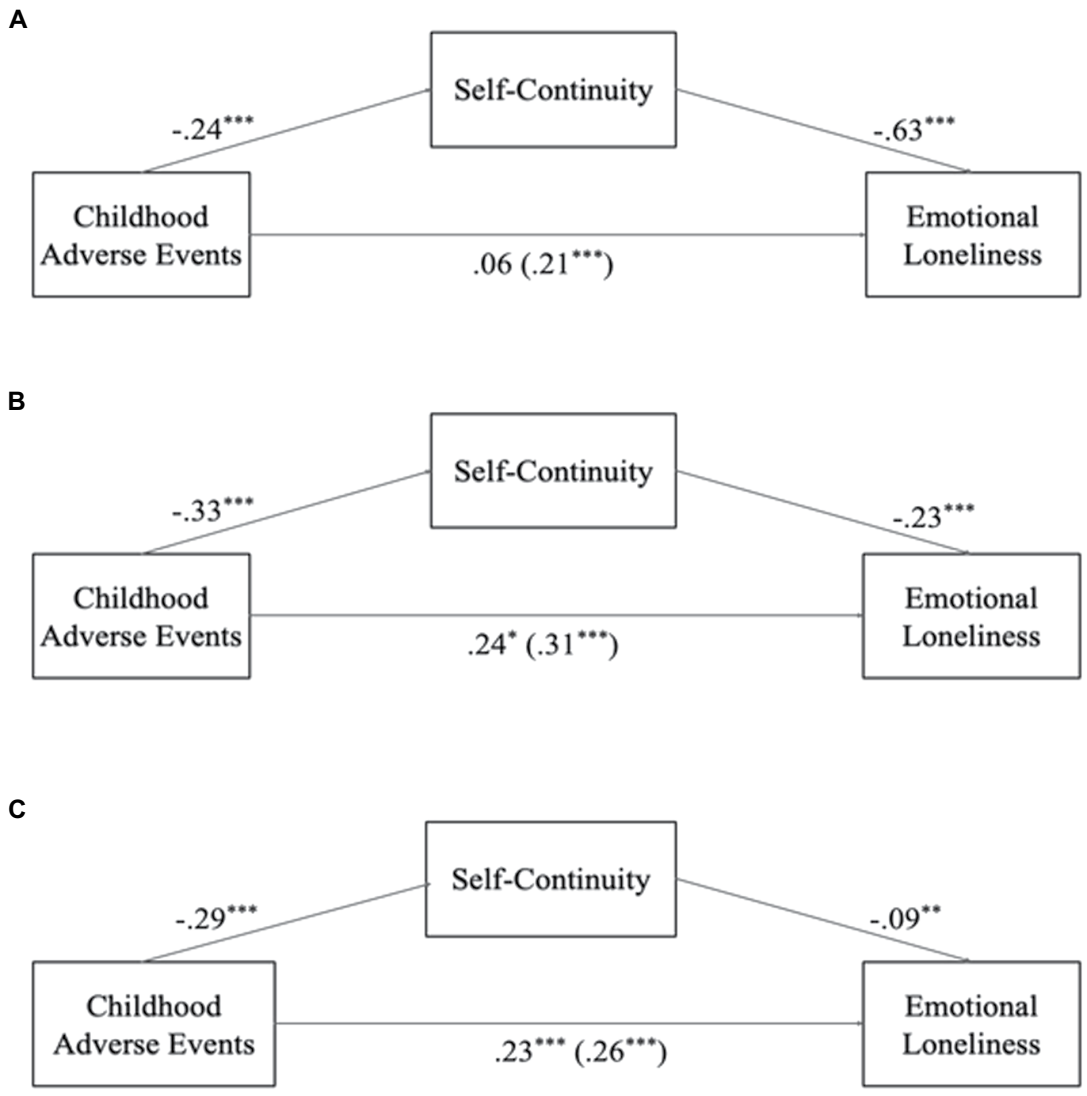
Between-subjects’ mediation patterns of self-continuity on the link between childhood adversity and emotional loneliness. Panel **(A)** = divorcees, indirect effect *B* = 0.15, *SE* = 0.04, 95% MCCI (0.071, 0.234), *z_sobel_* = 3.58, *p* < 0.001; Panel **(B)** = widowed, indirect effect *B* = 0.07, *SE* = 0.02, 95% MCCI (0.033, 0.124), *z_sobel_* = 3.14, *p* < 0.01; Panel **(C)** = married individuals, indirect effect *B* = 0.03, *SE* = 0.01, 95% MCCI (0.008, 0.048), *z_sobel_* = 2.51, *p* < 0.05; ^+^*p* < 0.10; *p < 0.05; **p < 0.01; ***p < 0.001.

For emotional loneliness, the first model (Figure 2B) revealed that self-continuity channeled the effect of childhood adversity on emotional loneliness (*B* = 0.19, *SE* = 0.03, 95% MCCI [0.135, 0.239], *Z_sobel_* = 7.03, *p* < 0.05). The moderation effect of marital status on the link between self-continuity and emotional loneliness was significant (*B* = −0.07, *p* < 0.05), indicating that divorced, widowed, and married individuals differed regarding the tested mediation. In the second model for emotional loneliness (Figure 3B), we once again added the loss-related covariates, and we found that a mediation process existed (*B* = 0.13, *SE* = 0.03, 95% MCCI [0.080, 0.186], *Z_sobel_* = 4.93, *p* < 0.001). However, this process differed in magnitude for the divorced and bereaved individuals, based on the significant moderating effect (*B* = −0.16, *p* < 0.01). Based on these first two models and in order to identify for which marital status the mediation effect was stronger, we ran separate mediation models for the divorced, widowed, and married groups. We found a full mediation of self-continuity on the link between childhood adversity and emotional loneliness for the divorcees (Figure 4A; *B* = 0.15, *SE* = 0.04, 95% MCCI (0.071, 0.234), *z_sobel_* = 3.58, *p* < 0.001), suggesting that divorced individuals who had experienced childhood adversity had lower levels of later-life self-continuity, and in turn, they felt more emotionally lonely. We also found partial mediations for the widowed and married groups (Figures 4B,C, respectively) with the same direction of effects [Widowed: *B* = 0.07, *SE* = 0.02, 95% MCCI (0.033, 0.124), *z_sobel_* = 3.14, *p* < 0.01; Married: *B* = 0.03, *SE* = 0.01, 95% MCCI (0.008, 0.048), *z_sobel_* = 2.51, *p* < 0.05]. Taken together, findings suggest that the link between childhood adversity and emotional loneliness were channeled by self-continuity in all marital status groups, but the indirect effect was particularly strong for divorced individuals.

### Covariate effects

In the following, covariate effects are described of the above-mentioned models, first describing models of social, and then emotional loneliness.

#### Between-subjects fixed effects for social loneliness

The results of Models 1 and 2 of the moderated mediations did not differ regarding the fixed effects of the covariates for social loneliness, with one exception: the presence of a new partner, which was added only in Model 2 (Table 2). Specifically, men (Model 1: *B* = −0.17, *p* < 0.001; Model 2: *B* = −0.28, *p* < 0.001), individuals with fewer financial resources (Model 1: *B* = −0.16, *p* < 0.001; Model 2: *B* = −0.11, *p* < 0.05), and people with fewer important social groups (Model 1: *B* = −0.16, *p* < 0.001; Model 2: *B* = −0.14, *p* < 0.001) felt more socially lonely. A less difficult childhood (Model 1: *B* = 21, *p* < 0.001; Model 2: *B* = 0.18, *p* < 0.001) and greater self-continuity (Model 1: *B* = −0.12, *p* < 0.05; Model 2: *B* = −0.13, *p* < 0.05) related to less social loneliness, confirming our expectation that self-continuity would be significantly and negatively associated to social loneliness. When having re-partnered was added in Model 2, this variable related significantly to social loneliness, suggesting that individuals who had not found new partners also felt more socially lonely (*B* = −0.33, *p* < 0.001). This finding indicated that even though social loneliness is more related to the unmet need for social embeddedness, having a new romantic partner has also a direct effect in feeling less socially lonely. Marital status and its interaction with self-continuity yielded no significant results. This result did not confirm our hypothesis that the link between self-continuity and social loneliness would be related to marital status and suggests that the relationship between the two constructs is independent of the experience of partner loss.

**Table 2:** Multilevel models with fixed and random effects of within- and between-subjects covariates and interactions on social loneliness.

	Model 1		Model 2	
	Divorced versus widowed versus married (*N* = 1,680)		Divorced versus widowed (*N* = 755)	
	Estimate	*SE*	Estimate	*SE*
*Fixed between-subjects’ effects*				
Age	−0.003	0.002	−0.01^+^	0.01
Gender (1 = women)	−0.17***	0.05	−0.28***	0.07
Financial adequacy	−0.16***	0.03	−0.11*	0.05
Time since event	-	-	0.01	0.02
Number of important groups	−0.16***	0.03	−0.14***	0.04
New partner (0 = no)	-	-	−0.33***	0.09
Childhood adverse events	0.21***	0.04	0.18***	0.05
Self-continuity	−0.12*	0.04	−0.13*	0.05
Marital status	0.11	0.09	0.08	0.18
Self-continuity*Marital status	−0.02	0.03	−0.06	0.07
*Fixed within-subjects’ effects*				
Intercept	1.65***	0.21	1.99***	0.39
Age	−0.03***	0.01	−0.04^+^	0.02
Number of important groups	−0.01	0.02	−0.03	0.02
New partner	-	-	−0.09	0.06
Self-continuity	0.01	0.04	−0.05	0.05
Self-continuity*Marital status	−0.003	0.03	0.08	0.07
*Random effects*				
Intercept self-continuity	0.52***	0.03	0.81***	0.05
Intercept social loneliness	0.78***	0.04	0.59***	0.04
Slope self-continuity	0.04*	0.01	0.05**	0.02
Residual variance social loneliness	0.30***	0.01	0.32***	0.01
Residual variance self-continuity	0.30***	0.01	0.33***	0.01
AIC	15173.08		8942.24	
−2LL (df)	15121.08 (26)		8878.24 (32)	
*ρ*	0.72		0.65	

Marital status = Divorced versus widowed versus married, or divorced versus widowed. df, degrees of freedom; AIC; Akaike information criterion; −2LL, −2 log likelihood, relative model fit statistics; ρ, intraclass correlation coefficient. Unstandardized estimates and standard errors are presented. ^+^*p* < 0.10; *p < 0.05; **p < 0.01; ***p < 0.001.

#### Within-subjects fixed effects for social loneliness

Regarding within-subjects fixed effects, as individuals grew older, they also experienced less social loneliness, with one unit of increase in age (i.e., a one-year increase) being associated to a decrease in social loneliness: Model 1, *B* = −0.04, *p* < 0.001 (Table 2; the effect became marginal in Model 2). The change in number of important groups, in self-continuity or the interaction between changes in self-continuity and marital status did not relate to changes in social loneliness in Model 1. Similar non-significant results were found for the change in partnership status and social loneliness in Model 2. Taken together, these findings showed that social loneliness is decreasing with age and was not associated with changes in other social- or identity-related processes.

#### Random effects for social loneliness

Regarding random effects, in Models 1 and 2, the intercepts of social loneliness (*B* = 0.52, *p* < 0.001; *B* = 0.81, *p* < 0.001, respectively) and of self-continuity (Model 1, *B* = 0.78, *p* < 0.001; Model 2, *B* = 0.59, *p* < 0.001) varied significantly, indicating that individuals differed regarding their average levels of social loneliness and self-continuity. In addition, we found a significant slope for self-continuity (Model 1, *B* = 0.04, *p* < 0.05; Model 2, *B* = 0.05, *p* < 0.01), indicating that self-continuity changed differently from one person to another. Finally, the within-subjects residual variances for social loneliness (Model 1, *B* = 0.30, *p* < 0.001; Model 2, *B* = 0.32, *p* < 0.001) and self-continuity (Model 1, *B* = 0.30, *p* < 0.001; Model 2, *B* = 0.33, *p* < 0.001) were also significant, indicating that individuals experienced significant changes regarding these variables with time.

#### Between-subjects fixed effects for emotional loneliness

Based on the significant moderating effects of marital status on the mediations described above for emotional loneliness (for detailed results on covariates see Appendix 1), we ran separate mediation models for each marital group. For the between-subjects effects, we found that older married individuals felt more emotionally lonely than their younger counterparts (*B* = 0.01, *p* < 0.05; Table 3, Model 3). For divorced people (Table 3, Model 1), lesser financial adequacy was related to higher emotional loneliness levels (*B* = −0.14, *p* < 0.05). Both divorced (*B* = −0.26, *p* < 0.001) and married individuals (*B* = −0.12, *p* < 0.001) with fewer important social groups felt more emotionally lonely; divorcees (*B* = −0.57, *p* < 0.001) and widowers (Table 3, Model 2; *B* = −0.26, *p* < 0.05) with new partners experienced lower levels of emotional loneliness, in line with theory regarding the influence a romantic partner for feeling less emotionally lonely. In addition, childhood adversity was negatively linked to emotional loneliness for widowed (*B* = 0.24, *p* < 0.001) and married individuals (*B* = 0.23, *p* < 0.001), but not for divorcees (*B* = 0.06, *p* > 0.05). The strong indirect effect on the mediation model for the divorcees explained the absence of this effect. Lower levels of self-continuity were related to feeling more emotionally lonely in all groups (divorced, *B* = −0.63, *p* < 0.001; widowed, *B* = −0.23, *p* < 0.001; married, *B* = −0.09, *p* < 0.01), but the effect was significantly stronger for the divorcees, as also evidenced by the indirect mediation effect.

**Table 3:** Multilevel models with fixed and random effects of within- and between-subjects covariates on emotional loneliness.

	Model 1		Model 2		Model 3	
	Divorced (*n* = 416)		Widowed (*n* = 339)		Married (*n* = 925)	
	Estimate	*SE*	Estimate	*SE*	Estimate	*SE*
*Fixed between-subjects’ effects*						
Age	0.003	0.01	0.001	0.01	0.01*	0.002
Gender (1 = women)	−0.22^+^	0.12	−0.09	0.09	0.07	0.05
Financial adequacy	−0.14*	0.06	−0.1	0.07	−0.04	0.04
Time since event	−0.03	0.03	−0.03	0.03	-	-
Number of important groups	−0.26***	0.06	−0.06	0.04	−0.12***	0.03
New partner (0 = no)	−0.57***	0.13	−0.26*	0.13	-	-
Childhood adverse events	0.06	0.07	0.24***	0.06	0.23***	0.04
Self-continuity	−0.63***	0.04	−0.23***	0.04	−0.09**	0.03
*Fixed within-subjects’ effects*						
Intercept	2.90***	0.53	1.51***	0.44	0.19	0.21
Age	−0.02	0.04	0.03	0.03	0.01	0.01
Number of important groups	−0.04	0.03	0.02	0.03	0.03	0.02
New partner	−0.28***	0.07	−0.45***	0.1	-	-
Self-continuity	−0.02	0.04	−0.01	0.04	−0.001	0.03
*Random effects*						
Intercept emotional loneliness	0.86**	0.29	0.67***	0.07	0.61***	0.04
Intercept self-continuity	0.88***	0.07	0.35***	0.04	0.23***	0.02
Covariance of Intercepts	0.58*	0.26	-	-	-	-
Residual variance emotional loneliness	0.31***	0.02	0.26***	0.02	0.17***	0.01
Residual variance self-continuity	0.34***	0.02	0.32***	0.02	0.25***	0.01
AIC	4991.55		3634.45		5273.3	
−2LL (df)	4933.55 (29)		3578.45 (28)		5229.30 (22)	
ρ	0.74		0.72		0.78	

df, degrees of freedom; AIC, Akaike information criterion; −2LL, −2 log likelihood, relative model fit statistics; ρ, intraclass correlation coefficient. Unstandardized estimates and standard errors are presented. ^+^*p* < 0.10; *p < 0.05; **p < 0.01; ***p < 0.001.

#### Within-subjects fixed effects for emotional loneliness

For within-subjects effects, when divorced (*B* = −0.28, *p* < 0.001) and widowed individuals (*B* = −0.45, *p* < 0.001) had a positive change in re-partnering status (i.e., they did not have a partner, but with time they found one), their levels of emotional loneliness significantly decreased: an increase in partnership status (here represented as having re-partnered) was associated with a decrease in emotional loneliness by 2.8 and 4.5 standardized units for the divorced and bereaved groups, respectively. No other within-subjects effect was observed for emotional loneliness, indicating that advancing to older age, increasing the number of important social groups, or increasing self-continuity were not related to changes in emotional loneliness.

#### Random effects for emotional loneliness

For the random effects, we observed significant variability in mean levels of emotional loneliness between individuals as indicated by the random intercepts (divorced, *B* = 0.86, *p* < 0.01; widowed, *B* = 0.67, *p* < 0.001; married, *B* = 0.61, *p* < 0.001). We found similar results for the random intercepts of self-continuity across groups (divorced, *B* = 0.88, *p* < 0.001; widowed, *B* = 0.35, *p* < 0.001; married, *B* = 0.23, *p* < 0.001). In addition, we found a significant covariance between the random intercept of emotional loneliness and the random intercept of self-continuity for divorced people (*B* = 0.58, *p* < 0.05), indicating that the more the intercept of emotional loneliness varied from the mean, the more the intercept of self-continuity varied. This covariance effect was only calculated for divorced individuals. This effect was not significant among the other two groups; it did not improve the fit of the model and was, therefore, excluded from the models presented. The within-subjects random variance for emotional loneliness was significant in all groups, indicating that individuals varied across measurement points regarding participants’ average levels of emotional loneliness (divorced, *B* = 0.31, *p* < 0.001; widowed, *B* = 0.26, *p* < 0.001; married, *B* = 0.17, *p* < 0.001). We further identified similar findings for the within-subjects random variance of self-continuity (divorced, *B* = 0.34, *p* < 0.001; widowed, *B* = 0.32, *p* < 0.001; married, *B* = 0.25, *p* < 0.001).

## Discussion

Addressing an important gap in the literature, our study investigated whether self-continuity mediates the link between childhood adversity and social and emotional loneliness, and whether these effects are moderated by marital status in the context of later-life divorce and bereavement. We found similar mediational patterns across divorced, widowed, and married (i.e., control) groups for social loneliness and differential mediational patterns for the marital status groups for emotional loneliness. Self-continuity mediated the link between childhood adversity and social loneliness, and marital status did not moderate these effects. This indicated that regardless of whether one had experienced the loss of a partner in later life, self-continuity channeled the effect of childhood adversity on social loneliness.

Regarding emotional loneliness, marital status moderated the mediation effect, revealing differential mediational patterns for each marital status group: for the divorced group, self-continuity fully mediated the link between childhood adversity and emotional loneliness. For the other two groups, the mediation effects were weaker. These findings, confirm that the experience of partner loss differs for divorce and widowhood (Pudrovska and Carr, 2008) and that childhood adversity has a greater impact on self-continuity in later life for divorcees, which associates to feeling significantly more emotionally lonely than the bereaved and married individuals. To our knowledge, this study is the first to confirm the mediating role of this identity process, linking distal life events with later-life adaptation to partner loss outcomes.

### Self-continuity associates with feeling less lonely

Our study underscores the important role of identity processes in older age. Individuals with a stronger perception of self-continuity felt less socially and emotionally lonely. We observed these findings for divorced and bereaved people, but also for married individuals, who served as the control group of this study. However, these results differed depending on the outcome and on whether individuals had lost their partners through divorce or bereavement. Regardless of whether they had lost an intimate partner, self-continuity helped individuals feel less socially lonely. These findings partly confirm our expectation that self-continuity and social loneliness would be related but this relationship did not differ by marital status. Self-continuity played a significant role in mediating the effect of childhood adversity on emotional loneliness as well, especially for divorcees. These findings are in line with previous research indicating that adaptation to partner loss differs between those who experienced a divorce and a partner’s death (Pudrovska and Carr, 2008). Divorcees may have to face feelings of disappointment and failure regarding the end of their marriage, making them question who they really are, but at the same time they may have a sense of control and agency over the event, in contrast to individuals confronted with spousal death. Although divorce is considered a negative life event, our findings suggest that it can also be an opportunity for more social ties than in widowhood or in marriage, similar to other negative life events (Haslam et al., 2008; Jones et al., 2012), either through social group memberships or *via* a new romantic partner. These findings may be particularly important for the development of self-continuity and its link to emotional loneliness for divorcees. Being an identity process that develops throughout the life course and with the property of fully channeling the negative influence of childhood adversity on emotional loneliness, self-continuity may get reinforced by multiple social group memberships and by the presence of a new romantic partner in a later adaptation phase, especially if the person felt closer to who they were before the break-up. Longer prospective studies with smaller between-waves intervals may be able to capture the beneficial effect of increased social engagement for self-continuity, which would then link the increasing sense of self-continuity to a decreasing feeling of emotional loneliness after divorce. In addition, it is of note that an experimental design aiming at reinforcing self-continuity [e.g., through nostalgia (Sedikides et al., 2015)] and social continuity (e.g., recalling important social groups) may allow examining causality between childhood adversity, self-continuity and loneliness. Given that this is the first longitudinal study to identify the beneficial relationship between self-continuity and loneliness dimensions after critical life events, our findings add to a better theoretical understanding of the usefulness of self-continuity as an identity resource in critical times. As this beneficial relationship remained even after controlling for other event-related factors (i.e., time since event) or lack of resources (i.e., financial inadequacy, few social groups, no repartnering) that could make individuals adapt better or faster, our findings may be valuable when applied in a therapy context. This can be also supported by the within-subjects effects we identified, that the increase in self-continuity levels was not significantly associated to a decrease in loneliness. These findings may indicate that the age-associated increase of self-continuity found by Lampraki et al., (2022) may not be sufficient to decrease loneliness. Therefore, the help of mental health experts may be needed in times of critical life events to further enhance this age-normative increase of self-continuity, through the restoration of autobiographical memory and the use of nostalgia (Jiang et al., 2020), which may lead to a decrease of emotional loneliness.

Our findings also point out that self-continuity is important for loneliness not only in times of crisis, including partner loss, but also in long-term marriages, adding to the literature regarding the positive effects of self-continuity on well-being. Long-term married individuals may benefit even more from self-continuity than divorcees, as they experience stability in their personal domain for a long period of time, which leads to more positive well-being outcomes later in life.

### Childhood adversity related to more loneliness but to a lesser extend when self-continuity was considered

Apart from proximal event-related factors and resources, we aimed at investigating the distal effect of childhood adversity on later-life social and emotional loneliness. In all groups, greater childhood adversity was related to lower levels of self-continuity and to experiencing more loneliness in later life. However, when self-continuity was added into the models, the relationship with the loneliness outcomes weakened or even disappeared. These findings add to the research regarding the role of self-continuity in channeling the effects of childhood adversity on loneliness. Bowlby (1951) had proposed that a lack of attachment in early life leads to loneliness in later life. Our findings not only highlight these connections but also suggest that the links are not straightforward, proposing an identity process as the mediator. As the first study to investigate this relationship in the context of a life crisis (e.g., divorce) or in marriage longitudinally, our results focus on the importance of identity processes for the long-lasting effects of childhood adversity on adaptation outcomes (Turner and Lloyd, 1995; Grotevant et al., 2017; Markovitch et al., 2017).

For emotional loneliness, divorcees with higher self-continuity levels than the population average felt less emotionally lonely and childhood experiences were no longer associated with emotional loneliness. This indirect influence of childhood adversity seems particularly important, as divorcees felt more emotionally lonely than the widowed and married groups and had overall experienced more childhood adversity. In line with previous research (Whisman, 2006; McCarthy and Maughan, 2010), these findings indicate that losing an intimate partner through divorce may be especially difficult for people who experienced adversity earlier in life, as their identity is also affected. Therefore, emotional loneliness in divorce seems to be more strongly related to whether individuals perceive discontinuity with regard to past selves than the actual experience of adversity in childhood, pointing to the need of interventions that reinforce identity in later life. These results add to previous research on adaptation to divorce: Loneliness is influenced not only by distal and proximal critical life events, but also by identity processes (Lampraki et al., 2019). Similarly in widowhood, individuals who experienced greater self-continuity felt less emotionally lonely, and childhood adversity had a smaller impact on their emotional loneliness levels.

### The role of age, social continuity and repartnering on social and emotional loneliness

The differences we observed among the marital status groups were also evident for the other predictors of emotional loneliness included in the models, such as age and number of important groups. For instance, older age was related to higher emotional (and social) loneliness only for married people, in line with previous research showing that poor marital quality in later life is related to emotional (and social) loneliness (de Jong Gierveld et al., 2009). Moreover, having fewer important social groups was related to greater emotional loneliness among divorced and married people, and to greater social loneliness for all marital status groups. These findings point out that social continuity is particularly important for social loneliness, in line with Weiss (1973) theory, but it is also contributing to feeling more emotionally lonely, over, and above not having a romantic partner. Not finding this effect for widowed individuals may be related to the fact that, in our sample, bereaved individuals were older than their divorced counterparts, with socioemotional selectivity theory suggesting that older age is related to less but more meaningful social relationships (Carstensen, 1992). In addition, we identified that changes as well as level differences in repartnering status associated to social and emotional loneliness. For instance, individual decline in emotional loneliness was associated with having found a new partner among divorcees and widow(er)s.

### Limitations

Despite our contributions, there are some limitations worth mentioning. The longitudinal nature of the large dataset allowed us to follow participants during their adaptation to partner loss. However, only a small proportion of our sample experienced the actual partner loss during the study, making it impossible to investigate pre- and post-loss levels of self-continuity and loneliness. Another limitation of our study is that we could not consider whether individuals had experienced other critical life events in their life courses, such as chronic illnesses or job losses that may have influenced self-continuity, as they were not part of the measures included in the full study. In addition, we had some attrition between our study and the full study sample related to the childhood adversity measure that was assessed only in the third wave and to the inclusion of participants that were older than 45 years of age at baseline, in order to have comparable partner-loss groups. Lastly, even though the theoretical justification for the direction of the effects has been described in extent in the introduction, we were not able to test causality between the different constructs. This is one limitation of the observational data, and these relationships must be tested in the future with an experimental design, reducing the bias from other possible factors not considered in our work.

## Conclusion

In conclusion, critical life events such as divorce and bereavement can be considered as normative in our societies, but they still relate to increased vulnerability in later life. However, when we consider that some individuals also carry childhood traumas, their vulnerability increases even more, as their identity is also impacted. Indeed, we found that self-continuity mediates the link between childhood adversity and loneliness. However, the extent to which it mediates this link depends on the type of loss experienced and the specific loneliness dimension. Divorced people seem to be more affected by childhood adversity than bereaved or married individuals, which determines the extent to which they benefit from self-continuity in times of crisis. Moreover, regardless of whether they have experienced a loss in the past, individuals who feel greater self-continuity also experience less social loneliness, and divorced individuals with a weak perception of self-continuity are significantly more emotionally lonely than bereaved and married individuals. This differentiation regarding facets of loneliness and events experienced offers guidance to mental health professionals on how to intervene to reinforce self-continuity when individuals are struggling with loneliness after partner loss in later life. Particularly in divorce, supporting individuals to find or reinforce emotionally meaningful relationships will promote identity stability, and therefore alleviate emotional loneliness. Although the evidence show that early life adversity maintains a strong influence on the lives of older adults, the fact that identity seems to channel its effects on well-being, is a positive result, as mental health professionals are directed toward reinforcing self-continuity after partner loss. Finally, later-life self-continuity is an identity process that is affected by childhood adversity, reinforcing the notion that distal childhood traumas continue to influence different facets of identity and well-being in later life, and that such traumas must be addressed early in the life course to promote resilience in later life.

## Data availability statement

The original contributions presented in the study are included in the article/Supplementary material, further inquiries can be directed to the corresponding author.

## Ethics statement

Approval for the study was obtained from the ethics committee of the University of Bern (2011-10-3864). The patients/participants provided their written informed consent to participate in this study.

## Author contributions

CL, DJ, and DS contributed to the conception and design of the study. CL organized the database, performed the statistical analysis, and wrote the first draft of the paper. DJ and DS revised the first draft of the manuscript. All authors contributed to the final version of the manuscript, read and approved the submitted version.

## Funding

This work was supported by the Swiss National Centre of Competence in Research LIVES – Overcoming vulnerability: Life course perspectives, which is financed by the Swiss National Science Foundation (grant number: 51NF40-160590). The authors are grateful to the Swiss National Science Foundation for its financial assistance.

## Conflict of interest

The authors declare that the research was conducted in the absence of any commercial or financial relationships that could be construed as a potential conflict of interest.

## Publisher’s note

All claims expressed in this article are solely those of the authors and do not necessarily represent those of their affiliated organizations, or those of the publisher, the editors and the reviewers. Any product that may be evaluated in this article, or claim that may be made by its manufacturer, is not guaranteed or endorsed by the publisher.
