# Supplementary material for: The mediating role of self-continuity on the link between childhood adversity and loneliness in later life
Source: Front Psychol. 2022 Nov 21;13:1039504. doi: 10.3389/fpsyg.2022.1039504 (PMC9721115; doi:10.3389/fpsyg.2022.1039504)
Supplement: Supplementary file 1 [file Table_1.DOCX]

**Appendix 1**

| Table 1. Descriptive Statistics of Study Variables by Study Wave | | | | | |
| --- | --- | --- | --- | --- | --- |
|  | Wave 1 (*n* = 1680) | Wave 2 (*n* = 1415) | Wave 3 (*n* = 1323) |  |  |
|  | *M* (SD) or *N* (%) | *M* (SD) or *N* (%) | *M* (SD) or *N* (%) | *F or χ^2^* | *p-value* |
| Social Loneliness | 0.96 (0.98) | 0.89 (0.96) | 0.87 (0.93) | 3.13 | .044 |
| Emotional Loneliness | 0.75 (0.85) | 0.78 (0.88) | 0.75 (0.84) | 0.66 | .518 |
| Age | 65.22 (11.56) | 66.26 (11.24) | 67.89 (11.04) | 20.67 | <.001 |
| Gender (women) | 974 (58) | 849 (60) | 794 (60) | 1.76 | .415 |
| Financial Adequacy | 2.06 (0.46) | 2.09 (0.47) | 2.11 (0.47) | 3.07 | .047 |
| Time Since Event | 2.80 (1.52) | 4.80 (1.52) | 6.80 (1.52) | 1315.78 | .000 |
| Number of important groups | 0.62 (0.98) | 0.50 (0.91) | 0.85 (1.04) | 33.70 | <.001 |
| New Partner | 0.64 (0.48) | 0.32 (0.47) | .60 (0.49) | 114.25*** | <.001 |
| Childhood adversity | - | - | 1.59 (0.68) | - | - |
| Self-continuity | 2.47 (1.08) | 2.42 (1.08) | 2.66 (1.11) | 16.39*** | <.001 |
| *Notes.* Time Since Event and New Partner apply only to divorced and widowed individuals. *χ^2^* test was performed only for Gender. | | | | | |

**Between Subject Fixed Effects**

The covariates’ effects did not differ between the first two moderated mediation models regarding their relationships to emotional loneliness, except for age and new partnership status. Specifically, in Model 1, older individuals felt more emotionally lonely (B = .01, *p* < .001) than their younger counterparts, but this effect was not present in Model 2 (see Table 3), indicating that only for divorced and bereaved individuals age did not account for differences in emotional loneliness. In both moderated mediation models, we found that poorer financial adequacy (Model 1, B = −.11, *p* < .001; Model 2, B = −.14, *p* < .05), fewer important social groups (Model 1, B = −.13, *p* < .001; Model 2, B = −.14, *p* < .001), greater childhood adversity (Model 1, B = .11, *p* < .01; Model 2, B = .10, *p* < .05) and less self-continuity (Model 1, B = −.59, *p* < .001; Model 2, B = −.64, *p* < .001) related to a higher levels of emotional loneliness later in life. In Model 2, having a new partner correlated to less emotional loneliness (B = −.44, *p* < .001). Moreover, in both models the main effect of marital status on emotional loneliness was positive and significant (Model 1, B = .37, *p* < .001; Model 2, B = .40, *p* < .05), while the interactions between mean-level self-continuity and marital status on emotional loneliness were also significant (significant moderating effects; Model 1, B = −.07, *p* < .05; Model 2: B = −.16, *p* < .01).

**Within Subject Fixed Effects**

Positive changes in re-partnering status (e.g., not having a partner but finding one with time) were related to a reduction in emotional loneliness levels (B = −.35, *p* < .001) in Model 2. It is of note that the rest time-changing variables, such as age, number of important groups, self-continuity, as well as the interaction between self-continuity and marital status, showed not significant associations with emotional loneliness.

**Random Effects**

Regarding random effects, in Models 1 and 2, the intercepts of emotional loneliness (B = .51, *p* < .001; B = .63, *p* < .001, respectively) and of self-continuity (Model 1, B = .78, *p* < .001; Model 2, B = .81, *p* < .001) varied significantly, indicating that individuals had different average levels of social loneliness and of self-continuity. In addition, we found significant covariances between the random intercepts of emotional loneliness and self-continuity (Model 1, B = .33, *p* < .01; Model 2, B = .40, *p* < .05). This indicated that the more the mean level of emotional loneliness varied across individuals, the more the mean level of self-continuity varied across individuals, too. Finally, the within subject residual variances for emotional loneliness (Model 1, B = .25, *p* < .001; Model 2, B = .29, *p* < .001) and self-continuity (Model 1, B = .30, *p* < .001; Model 2, B = .34, *p* < .01) were also significant, indicating that individuals experienced significant changes regarding these variables with time.

| Table 2. Multilevel Models with Fixed and Random Effects of Within- and Between-Subjects Covariates and Interactions on Emotional Loneliness | | | | |
| --- | --- | --- | --- | --- |
|  | Model 1 | | Model 2 | |
|  | Divorced vs Widowed vs Married (*N* =1680) | | Divorced vs Widowed  (*n* =755) | |
|  | Estimate | SE | Estimate | SE |
| **Fixed Between-Subjects’ Effects** |  |  |  |  |
| Age | .01^***^ | .002 | .01 | .004 |
| Gender (1 = *women*) | .01 | .05 | -.15^+^ | .08 |
| Financial adequacy | -.11^***^ | .03 | -.14^*^ | .04 |
| Time since event | - | - | -.01 | .02 |
| Number of important groups | -.13^***^ | .03 | -.14^***^ | .04 |
| New partner (0 = *no*) | - | - | -.44^***^ | .09 |
| Childhood adverse events | .11^**^ | .03 | .10^*^ | .05 |
| Self-continuity | -.59^***^ | .04 | -.64^***^ | .05 |
| Marital status | .37^***^ | .08 | .40^*^ | .16 |
| Self-continuity*Marital status | -.07^*^ | .03 | -.16^**^ | .06 |
| **Fixed Within-Subjects’ Effects** |  |  |  |  |
| Intercept | 2.04^***^ | .18 | 2.47^***^ | .37 |
| Age | -.01^+^ | .01 | .002 | .02 |
| Number of important groups | .02 | .02 | -.01 | .02 |
| New partner | - | - | -.35^***^ | .06 |
| Self-continuity | -.04 | .04 | -.01 | .04 |
| Self-continuity*Marital status | -.01 | .03 | .03 | .06 |
| **Random Effects** |  |  |  |  |
| Intercept emotional loneliness | .51^***^ | .09 | .63^***^ | .15 |
| Intercept self-continuity | .78^***^ | .04 | .81^***^ | .05 |
| Covariance of Intercepts | .33^**^ | .13 | .40^*^ | .17 |
| Residual variance emotional loneliness | .25^***^ | .01 | .29^***^ | .01 |
| Residual variance self-continuity | .30^***^ | .01 | .34^***^ | .02 |
| AIC | 14376.91 | | 8632.46 | |
| -2LL (df) | 14324.91 (26) | | 8568.46 (32) | |
| ρ | .67 | | .68 | |
| *Notes:* Marital status = *Divorced vs Widowed vs Married, or Divorced vs Widowed.* df = *degrees of freedom.* AIC = *Akaike information criterion*; –2LL = *–2 log likelihood*, relative model fit statistics. ρ = *Intraclass Correlation Coefficient*. Unstandardized estimates and standard errors are presented. ^+^*p* < .10; **p* < .05; ***p* < .01; ****p* < .001. | | | | |
